# Cometabolism of Chlorinated Volatile Organic Compounds and 1,4-Dioxane in Groundwater

**DOI:** 10.3390/w15223952

**Published:** 2023-11

**Authors:** Catherine Clark, Lee K. Rhea

**Affiliations:** Subsurface Remediation Branch, Groundwater Characterization and Remediation Division, Center for Environmental Solutions and Emergency Response, U.S. Environmental Protection Agency, 919 Kerr Research Drive, Ada, OK 74820, USA

**Keywords:** groundwater, chlorinated volatile organic compounds, 1,4-dioxane, cometabolism

## Abstract

This article provides an overview of the bioremediation of groundwater plumes containing admixtures of chlorinated volatile organic compounds (CVOCs) and 1,4-dioxane. The remediation of these plumes has historically focused on the reductive dechlorination of the CVOCs. Many of the remaining plumes are relatively large, and contaminant concentrations are diluted below the concentrations that can sustain reductive dechlorination. Cometabolic processes can decrease contaminant concentrations below the thresholds needed to support direct metabolism but typically require the addition of a substrate, such as high-purity propane. Relatively intensive site characterization and monitoring is necessary to implement bioremediation.

## 1. Introduction

In the past, the bioremediation of groundwater plumes containing chlorinated volatile organic compounds (CVOCs) focused on their destruction under strongly reducing conditions through a bacterial metabolic process known as reductive dechlorination. This process reduces the concentrations of the CVOCs but rarely eliminates them entirely [1]. Reductive dechlorination also does not degrade a common co-contaminant, 1,4-dioxane (dioxane) [2,3]. Plumes of comingled CVOCs and dioxane typically originate from areas where either of the solvents tetrachloroethene (PCE) and trichloroethene (TCE) was used prior to being replaced by the somewhat less toxic and environmentally persistent solvent 1,1,1-trichloroethane (TCA). Dioxane is often present with these CVOCs because it was commonly added to TCA as a stabilizer [4]. Decades later, the remaining plumes of comingled CVOCs and dioxane became often large and diluted. Due to this, the remediation of these plumes is difficult and expensive due to chemical and hydrogeologic concerns [5]. Large and dilute plumes are informally defined as being miles in length [6] and have contaminant concentrations of less than 1 mg/L [7]. Large, dilute groundwater plumes containing comingled CVOCs and dioxane (LDCD hereafter) can extend miles and be very deep because they have often existed for decades. Such plumes commonly occur within aquifers that have high groundwater flow rates, are aerobic, and are low in organic carbon and biomass. As might be expected under these conditions, these plumes attenuate slowly [6].

**Figure 1.**
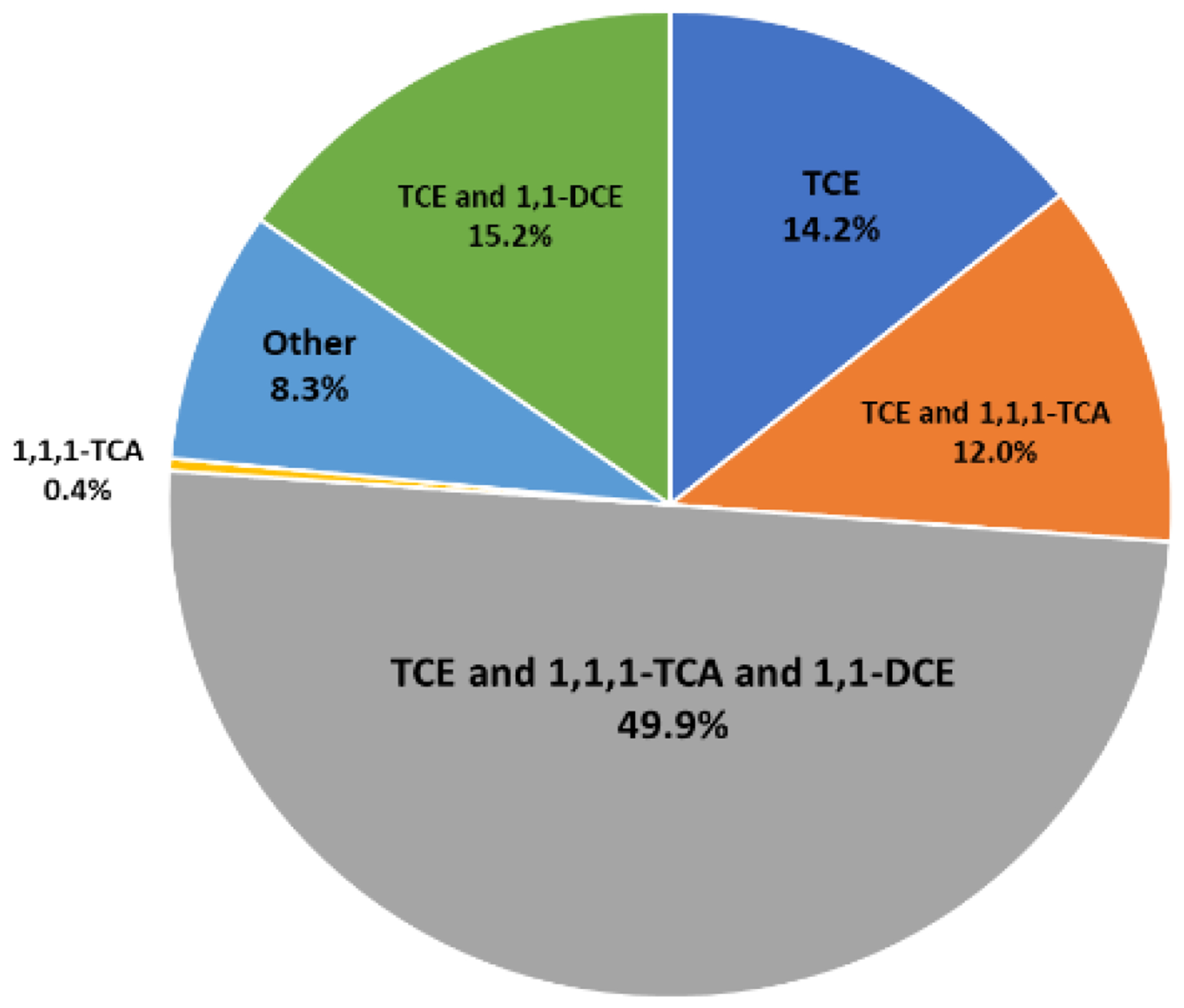
Groundwater co-contaminants present in 46 USAF installations that contain 1,4-dioxane. [4]. Used with permission.

A study examining 46 U.S. Air Force installations in which dioxane was detected found that half of them also contained TCE, TCA, and 1,1-dichloroethene (1,1-DCE) [4] (Figure 1), and the relative lengths of the dioxane and CVOC components were variable. The downgradient extents of the CVOCs in these plumes frequently increased in the order of PCE, TCE, DCEs, and vinyl chloride (VC), but the downgradient extent of comingled dioxane varied considerably [8]. Based on the data mining of more than 2000 sites in California, Adamson et al. (2015) [9] found that 62% of dioxane plume lengths were shorter than comingled CVOC plumes, and 21% of dioxane plumes were longer than comingled CVOC plumes. Differing relative lengths of dioxane versus CVOCs in plumes may result because dioxane is comparatively highly water-soluble and has a low affinity for retardation or volatilization [10,11]. Consequently, the source of the dioxane plume may have been more depleted than that of the CVOCs. Also, dioxane may degrade faster than CVOCs in some oxidizing groundwater environments, rendering the dioxane plume shorter.

The biotically mediated destruction of CVOCs and dioxane in groundwater usually occurs under different redox conditions and at higher contaminant concentrations than those in large dilute plumes. Studies have, however, demonstrated the feasibility of remediating plumes of comingled CVOC and dioxane by directly addressing these issues using a “treatment train” approach [7,12]. These studies initially employed reductive dechlorination under anaerobic conditions to degrade CVOCs; then, they oxygenated the water to degrade dioxane under aerobic conditions. In theory, this concept may work well for smaller-scale applications, but creating anaerobic conditions in a large plume and subsequentially reoxygenating it would be extremely expensive and impractical [6]. Further, forcing anaerobic conditions on aerobic groundwater can cause the dissolution of iron and sulfides, leading to the precipitation of iron hydroxides and formation of hydrogen sulfide [6]. Concurrent aerobic cometabolism may be a more cost-effective method of bioremediation for such plumes [13]. This method does not require redox manipulations when the concentrations of CVOCs and dioxane are low.

## 2. Biodegradation Pathways

The bioremediation of CVOC plumes in groundwater by both natural and engineered means has been well studied. The employment of biodegradation as a remedial technique for CVOC plumes has traditionally utilized reductive dechlorination, which requires anaerobic, highly reducing conditions in the sulfate-reducing or methanogenic ranges [14]. It has been known for decades that CVOC plumes can sometimes biodegrade via aerobic metabolism and cometabolism [15]. Reductive dechlorination is a metabolic process where bacteria use CVOCs as electron acceptors and require reducing conditions [16]. Chlorine atoms are sequentially removed from CVOCs during reductive dechlorination; under optimal conditions, CVOCs can be converted to ethane, although this process frequently stalls at the DCEs (known as the “DCE stall”) [16]. Bacteria that perform reductive dechlorination are obligate anaerobes and often favor heavily chlorinated VOCs such as PCE and TCE [17] because dechlorinating these generates more energy than degrading lightly chlorinated VOCs such as DCEs and VC [18]. This can result in an accumulation of DCEs [19]. The accumulating DCEs and VC are frequently referred to as daughter products [1]. However, there are a few bacterial strains within genera *Dehalococcoides* [20,21] and *Dehalogenimonas* [22] that can dechlorinate DCE and VC as well as PCE and TCE, but these bacteria may not have a sufficient population density to compete with other bacteria that perform reductive dechlorination [17]. Although anaerobic bioremediation has often been successful in degrading heavily chlorinated CVOCs in groundwater, it has not been as successful in treating plumes of comingled CVOCs and dioxane [7].

The biodegradation of dioxane in groundwater has also been well studied. CVOCs and dioxane are typically degraded by different bacteria and redox conditions. Other in situ remedial methods that may have been previously applied typically have limited success in removing or destroying both compounds at the same time [7]. It was previously thought to be difficult for microbes to metabolize dioxane due to its cyclic diether structure [23], consistent with dioxane often having a greater downgradient extent than comingled CVOCs. However, although dioxane biodegradation is still thought to be unlikely in anaerobic groundwater [24], it is now recognized that it can occur under aerobic conditions. Dioxane bio-attenuation can occur either co-metabolically or when it is used as the sole carbon and energy source [4].

Many of the enzymes that facilitate aerobic metabolism and cometabolism are soluble di-iron monooxygenases (SDIMOs), which are a family of nonheme bacterial enzymes. [25] Monooxygenases oxidize TCE, DCEs, and VC via both metabolic and cometabolic pathways into various metabolites such as epoxides and alcohols. These metabolites then enter the central metabolism, which ultimately degrades them to carbon dioxide [17]. Although dioxane metabolizers such as CB1190 and PH-06 typically have group-5 tetrahydrofuran (THF) MOs and group-6 propane monooxygenases, other cometabolizers of dioxane and CVOCs have a very wide range of monooxygenases such as toluene-2-MO, toluene-4-MO, and short chain alkane MO [25]. These include some bacterial strains that can degrade CVOCs and dioxane at the same time (Table 1). Cosubstrates for cometabolic degradation include ethane, butane, butanol, THF, propane, propanol, and toluene [25–28]. Nitrogen and phosphorus must also be present as nutrients. Cosubstrates not only provide a carbon source for growth but also induce necessary cometabolic enzymes.

## 3. Biodegradation Kinetics

The biodegradation of dioxane varies with the facilitating microbe and their microenvironment. A study by Barajas-Rodriguez et al. (2018) [31] measured Monod parameters to model the biodegradation kinetics of dioxane metabolizer CB1190 and dioxane cometabolizers *R. ruber* ENV 425 as well as a mixed consortium of propanotrophs ENV487. While CB1190 was found to have the highest maximum specific degradation rate, the cometabolic strains were able to degrade 1000-to-1 ppb dioxane in less time than CB1190. This is due to CB1190 needing dioxane as a carbon source to maintain its growth. This finding showed that dioxane degradation rates by CB1190 are more heavily influenced by initial biomass when compared to the degradation rates of cometabolizers ENV425 and ENV487 [31]. One caveat that was observed with cometabolism in this study is that propane metabolism competitively inhibited dioxane cometabolism. This can be mitigated by adding propane in cycles and alternating propane and oxygen injections.

Dioxane biodegradation was further investigated by Barajas-Rodriguez et al. (2019) [32] by coupling a steady-state air sparging flow model with a contaminant transport model along with Monod kinetics for dioxane metabolism and cometabolism. The coupled models were created using COMSOL 5.2 Multiphysics software. The geometry and parameters were developed from field data from a site at Vandenberg Air Force Base where bioaugmentation and propane bio sparging were used to degrade dioxane [31,32]. The simulation results showed that CB1190 was more effective at degrading dioxane at concentrations equal to or greater than 10 milligrams per liter (mg/L), but ENV425 outperformed CB1190 when dioxane concentrations were 7.5 mg/L or less. This is likely due to CB1190 not having enough carbon to sustain the same growth rate as ENV425. Considering that dilute mixed plumes are typically low in organic carbon and have contaminant levels around 100 μg/L [33], the findings of these studies support cometabolism as a better option for bioremediating these plumes.

## 4. Substrate Delivery

**Figure 2.**
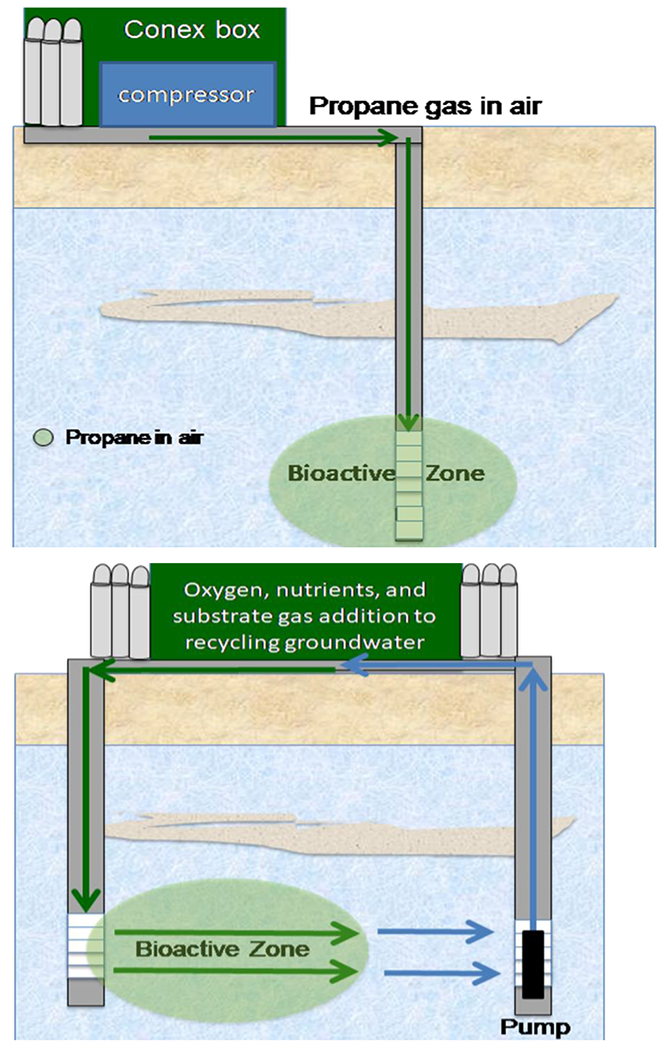
Graphical representation of active biosparging (**top**) and groundwater recirculation (**bottom**). Taken from [37]. Used with permission.

Cometabolic bioremediation frequently involves delivering a gaseous substrate into the subsurface. This can be accomplished with active biosparging and groundwater recirculation [1]. Active biosparging involves injecting air and gaseous substrates into ground water via a compressor. Active biosparging requires no pumping infrastructure (Figure 2), has a good radius of influence (ROI), and can be used on confined and unconfined aquifers, but a preferential flow path is required [1]. Active biosparging was used to remediate a site in Vandenberg Air Force Base near Santa Barbera, California. This site contained 1,4 dioxane as well as TCE, DCEs, and DCAs [34]. A field demonstration by Lippincott et al. (2015) [35] developed a biosparging system for this site using two existing monitoring wells, two new monitoring wells, and a new sparge well. The monitoring wells had initial dioxane concentrations between 113 and 1090 μg/L. They conducted microcosm studies for this site and determined that propanotrophs were present but not in high-enough numbers to readily degrade dioxane. Due to this finding, they decided to bioaugment with dioxane cometabolizer ENV 425 to shorten the lag time. Diammonium phosphate was also added as a nitrogen and phosphate source to also improve microbial growth. After a nine-month period, the dioxane concentrations in the sparge well and three of four monitoring wells were between <2 and 7.4 μg/L (2 μg/L is the practical quantitation limit [PQL]) [35].

Groundwater recirculation can also be used to pump gaseous substrates along with liquid amendments such as nutrients and cultures (Figure 2) [1]. In a field study by Chu et al. (2018) [36], a groundwater recirculation system was developed and operated in the McClellan Air Force Base in California. The contaminants of interest at this site were dioxane, 1,2-DCA, and TCE, which were present in concentrations of 59, 21, and 5.1 μg/L, respectively. This system used biostimulation to promote the degradation of these contaminants by the native microbial population by adding oxygen and consumer-grade propane (H10). Over a nine-month period, this system achieved treatment efficiencies of 91%, 96%, and 91% for TCE, dioxane, and 1,2-DCA, respectively. Biofouling can be an issue for these systems, but it can be mitigated by alternating substrate and oxygen additions, which creates a bioactive zone that is further away from the injection well [36]. Hydrogen peroxide can also be added to reduce biofouling and provide an additional source of oxygen. Chu et al. (2018) [36] added hydrogen peroxide to achieve approximately 100 mg/L in the recirculated groundwater.

## 5. Substrate Quality

The quality of the substrate can also be important along with the delivery method. Consumer-grade gaseous substrates contain impurities that can sometimes retard microbial growth. For example, consumer-grade propane can contain up to 10% propylene, which has been found to inhibit the growth of propanotrophs [1]. The metabolic oxidation of alkenes such as propylene generates epoxides, which are harmful to bacteria. While alkene metabolizers can sequester and neutralize epoxides, alkane metabolizers typically lack these detoxifying processes. A study by Meza (2020) [38] confirmed this by using R. rhodochronus ATCC 21198 as a model propanotroph and exposing it to propane mixtures containing propylene, ethane, and ethanethiol. They found that increasing the concentrations of propylene decreased growth rates and increased propylene oxide production. No inhibition was observed when increasing concentrations of ethane or ethanethiol were added. Field demonstrations, such as the groundwater recirculation system created by Chu et al. (2018) [36], have successfully used consumer-grade propane as a substrate for biostimulation. Meza (2020) [38] hypothesized that this may be due to native alkene metabolizers neutralizing the propylene oxide before it inhibits the propanotroph population. Chu et al. (2018) [36], Horst et al. (2019) [39], and Lippincott et al. (2015) [35] all stated that they used consumer-grade propane to increase microbial diversity and to potentially stimulate dioxane cometabolism that uses other substrates. Considering the outcomes of these studies, consumer-grade substrates can still be used, but if dioxane degradation is stagnating, then laboratory-grade propane should be considered, especially when bioaugmenting with bacteria that cannot metabolize alkenes.

## 6. Inhibitory and Stimulatory Factors

The inhibitory effects of CVOCs on dioxane metabolism have been documented [40]. A study by Zhang et al. (2017) [41] tested the biodegradation kinetics of dioxane metabolism in the presence of CVOCs using *Pseudonocardia dioxanivorans* CB1190 as a model dioxane metabolizer. CB1190 was grown with 0.5 mg/L dioxane and 0.5, 5, or 50 mg/L of 1,1-DCE, cis-DCE, TCE, or TCA. This study found that 1,1-DCE was the most inhibitory followed by cis-DCE, TCE, and then TCA. The effects of chlorinated solvents on dioxane cometabolism at environmentally relevant concentrations are less understood, however. There have been biodegradation kinetics studies that suggest that the inhibition of dioxane cometabolism by chlorinated solvents is likely due to product toxicity [27,30,42]. Another study by Li et al. (2020) [28] compared the dioxane degradation kinetics of propane and tetrahydrofuran monooxygenases (MO) and found that propane MO degrades dioxane faster, is less inhibited by chlorinated solvents, and has a greater substrate range. This study also found that the inhibition of both monooxygenase by 1,1-DCE and TCA is non-competitive, which is likely due to 1,1-DCE or TCA binding to an allosteric site. Inhibition by TCE is likely competitive for both enzymes.

Biodegradation kinetics studies often generate useful information, but they use substrate concentrations that are often ten times higher than what are present in LDCDs. However, the two field demonstrations previously mentioned both addressed dilute mixed plumes and were both fed propane to stimulate cometabolism for dioxane and chlorinated solvents [35,36]. The site used by Lippincott et al. (2015) [35] had 34, 411, 21, 286, 69, 30, and 226 μg/L of PCE, TCE, cis-1,2-DCE, 1,1-DCE, 1-1 dichloroethane (1,1-DCA), 1,1,1-TCA, and trichloroflouromethane, respectively. The site used by Chu et al. (2018) [36] had 21 ug/L of 1,2-DCA and 5.1 ug/L of TCE. Both studies reported that inhibition by chlorinated solvents was not an issue and that the chlorinated solvents present were degraded to some extent. With these findings in mind, using propane MO expressing microbes appears to be viable for treating dilute mixed plumes. However, further research is needed to confirm this and to better understand chlorinated solvent inhibition at the concentrations present in dilute plumes containing both CVOCs and dioxane.

In addition to substrates, bioremediation often requires the addition of nitrogen and phosphorus as micronutrients. For field-scale applications, these nutrients are typically added as diammonium phosphate, which is injected as a brine solution [35]. Dissolved metals also affect dioxane-degrading bacteria. Manganese (II) and iron (III) at concentrations of 0.001–0.1 mg/L and 0.5–10 mg/L, respectively, can be stimulatory while copper (II), cobalt (II), and higher levels of iron (III) can be inhibitory [43].

Lastly, the optimal temperature for dioxane biodegradation is 20 to 30 degrees Celsius (C), and temperatures below 4 degrees C are extremely inhibitory [44]. Temperature is an important factor to consider because many areas above the 50° N latitude, such as Canada and Northern Europe, have groundwater temperatures below 5 °C [45]. For more information, see Table 2.

## 7. Monitoring

Although bioremediation has been successful for the mitigation of CVOCs and dioxane, it requires more monitoring than other methods, such as pump and treat or chemical oxidation [46]. Such a remedy requires the demonstration of efficacy using multiple lines of evidence, including the estimation of biodegradation rate constants using a transport and fate model calibrated using site-specific data. Monitoring is necessary prior to bioremediation to determine whether suitable aquifer conditions exist or can be attained, during treatment to determine if suitable conditions are being maintained and remediation is occurring, and after treatment to document that satisfactory remediation has occurred.

The parameters that should be monitored vary by the phase of bioremediation and by the methods they should be assessed with (Table 2). Aquifer porosity, hydraulic conductivity, and groundwater flow gradients should be assessed prior to treatment because they influence transport through the plume of any added cometabolic microbes. There are several methods to obtain this information, but all estimates must be confirmed with on-site measurements made on undisturbed media via methods such as slug or pump tests.

Groundwater geochemical parameters including oxidation reduction potential (ORP), dissolved oxygen, carbon dioxide, and pH should be monitored before and during treatment to assess the suitability of groundwater chemistry for cometabolism [47]. These measurements should be measured in the field using portable field screening equipment. Although readily obtained, these data are only considered an indirect line of evidence for demonstrating biodegradation. Groundwater concentrations of contaminants and the primary substrate(s) should be measured before, during, and after treatment. These measurements are used before and during treatment to assess contaminant levels and ensure that adequate substrates are available for cometabolic microbes, and contaminant concentrations should be measured after treatment to document whether they have been adequately remediated. These measurements should be made using gas chromatography (GC) or GC combined with mass spectrometry (GC/MS) [47].

The presence and activity of microbes necessary for cometabolic bioremediation should be assessed both prior to and during treatment, but the available methods have significant limitations. The classes of available methods are Environmental Molecular Diagnostic (EMD) tools, Compound-Specific Isotope Analysis (CSIA), and carbon-14 (C^14^) assays. All these EMD tools are based on DNA extracts, and they include Quantitative Polymerase Chain Reaction (qPCR), Reverse Transcriptase qPCR (RT-qPCR), and microarrays. Additional information about EMD is summarized in Table 3.

CSIA uses the fractionation of stable isotopes to discern whether contaminant reduction in groundwater plumes is due to destructive processes or non-destructive processes such as dilution and dispersion. The concept underlying the Compound-Specific Isotope Analysis (CSIA) is that many atoms in the environment exist as mixtures of isotopes, and many natural processes that involve the movement or chemical reaction of atoms or molecules have characteristic propensities to act on lighter isotopes rather than heavier isotopes. Additional information about CSIA is summarized in Table 3.

An assay based on contaminant radiolabeling with C^14^ has recently been developed that may address limitations of EMD tools and CSIA [48–53]. This assay is available for several CVOCs and dioxane and is available from Microbial Insights Inc. It is applied to groundwater samples to detect biotically mediated destruction but requires both soil and groundwater samples to detect abiotic degradation. Bioremediation rate constants can be calculated using the results of the assay.

## 8. Summary

The bioremediation of groundwater plumes containing mixtures of CVOCs and 1,4-dioxane has historically focused on the reductive dechlorination of the CVOCs by microbiota, but this mechanism is unsuitable for the remaining plumes that also contain dioxane and are relatively dilute. Although the contaminant concentrations in such plumes are relatively low in comparison to many legacy plumes, the contaminants still sometimes remain above regulatory thresholds. Microbial cometabolism can degrade contaminants to concentrations below the thresholds needed to support direct metabolism and below regulatory thresholds but typically requires the addition of a substrate such as high-purity propane. Cometabolism experiments using a treatment-train approach with reducing conditions to degrade CVOCs followed by oxidizing conditions to degrade dioxane have been successful, as have experiments using an aerobic environment to simultaneously degrade both CVOCs and dioxane. Cometabolism may be the only economically feasible remedial method for large dilute plumes, but relatively intensive site characterization and monitoring is nonetheless necessary to implement bioremediation.

Several conclusions can be drawn from the available information:

The biochemistry of microbial cometabolism is complex and depends on multiple parameters, but suitable conditions for bioremediation via cometabolism are possible through biostimulation and bioaugmentation. Microcosm studies are needed to confirm on a site-specific basis what is needed to facilitate biodegradation. Finetuning a bioremediation strategy on a smaller scale is less costly over the lifecycle of a site remediation.

Multiple studies have shown that propane can be an optimal substrate for cometabolism. Propane monooxygenases can degrade a wider variety of contaminants and have a higher affinity for dioxane [27,28,30]. Propane is also less toxic and cheaper than other substrates.

**Figure 3.**
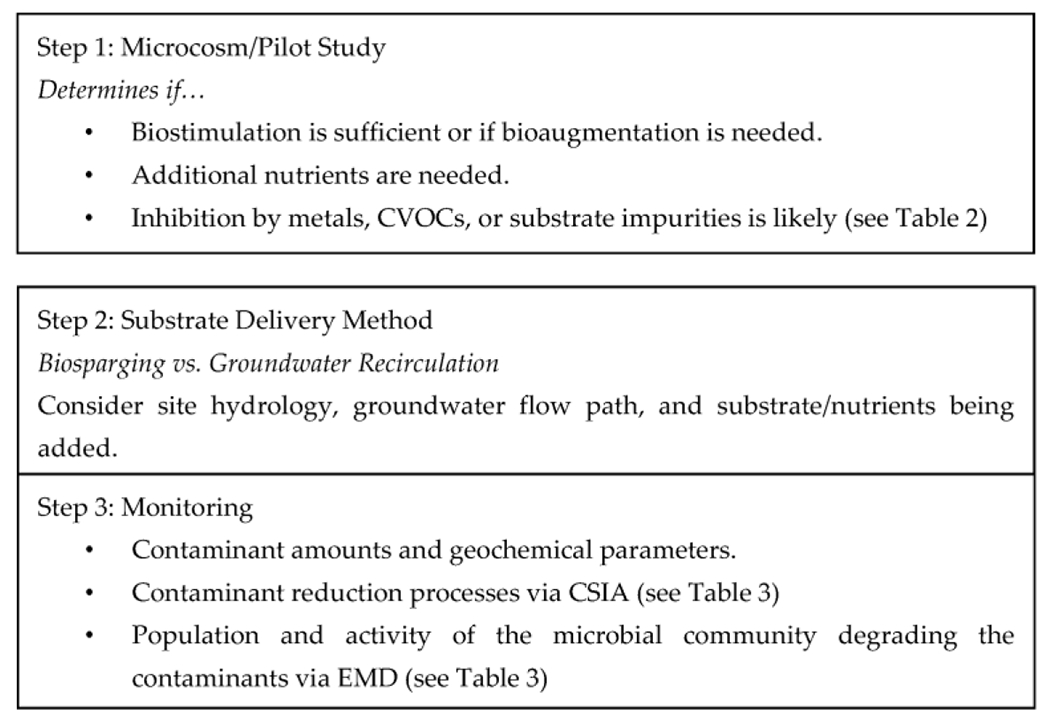
Graphical representation of the summary and recommendations.

Sites that contain dioxane concentrations of over 10 mg/L may be better remediated by augmenting with a dioxane metabolizer and cometabolizer. As stated earlier, metabolism is more effective at concentrations above 10 mg/L, and cometabolism is more effective at concentrations below 7.5 mg/L [31,32]. Having both will reduce stagnation when the concentration is reduced below 7.5 mg/L. For a graphical representation of the summary and recommendations made, see Figure 3.

## Figures and Tables

**Table 1.** List of bacterial strains and mixed consortiums that cometabolize dioxane and CVOCs.

Microbe/Mixed Culture	Contaminant	Substrate	References
*Azoarcus* sp. strain DD4	Dioxane, 1,1 DCE	Propane, Toluene, propanol	[25]
*Mycobacterium vaccae* JOB5	Dioxane, TCE, DCE, VC	Propane, butane, pentane, isobutane, isopentane,	[17,25]
*Mycobacterium sphagni* ENV482	Dioxane, TCE	Ethane	[26]
*Mycobacterium chubuense* strain NBB4	Dioxane, cis-DCE, 1,2-DCA, VC	C2-C4 alkenes, C2-C16 alkanes	[1]
*R. rhodochrous* ATCC 21198	Dioxane, TCA	2-butanol, propane	[29]
*Arthrobacter* sp. WN18	Dioxane, VC	THF	[30]
*Burkholderia cepacia* strain G4	Dioxane, TCE	Toluene	[17,25]
*Pseudomonas mendocina* strain KR-1	Dioxane, TCE	Toluene	[17,25]
*R. ruber* ENV 425	Dioxane	Propane	[31]
Mixed consortium of propanotrophs ENV487	Dioxane	Propane	[31]
*Mycobacterium* sp. ENV 421	Dioxane	Propane	[28]
*Pseudonocardia* sp. K1	Dioxane	THF	[28]
*Pseudonocardia* sp. ENV 478	Dioxane	THF	[28]
*Methylosinus trichosporium* strain OB3b	1,1-DCE, TCE	Methane	[17,25]
*Pseudomonas stutzeri* strain OX1	1,1-DCE, PCE	Toluene	[15,17,25]

**Table 2.** Factors that affect 1,4 dioxane cometabolism.

Factor	Stimulatory (a) or Optimal (b)	Inhibitory (c) or Suppressive (d)	References
oxygen	4–11 mg/L (b)	<1.5 mg/L (d)	[35,36]
Mn(II)	0.001–0.1 mg/L (a)		[43]
Cu(II)		>2 mg/L (c)	[43]
Co(II)		>5 mg/L (c)	[43]
Fe(III)	0.5–10 mg/L (a)	>50 mg/L (c)	[43]
1,1-DCE		>2–0.5 mg/L (c)	[28]
TCE		>1–0.5 mg/L (c)	[28]
1,1,1-TCA		>2 mg/L (c)	[28]
Temperature	20–30 ° C (b)	<4 °C (d)	[44]
Total Nitrogen		<50 ug/L (d)	[44]

Note: The letters on each data denote whether that factor is Stimulatory (a ) or Optimal (b) or Inhibitory (c) or Suppressive (d).

**Table 3.** EMD and CSIA descriptions and limitations [54]. Adapted from [54]; used with permission.

Tool	What It Detects	What It Determines	Limitations
Compound-Specific Isotope Analysis (CSIA)	Fractionation of heavier and lighter stable isotopes of a compound	- If contaminant reduction is due to destructive processes or non-destructive processes such as dilution and dispersion. - Direct indicator of degradation	- Degradation rate constants calculated using CSIA are often highly variable throughout a plume - Difficult to separate results from different degradation processes and different sources.
EMD: Quantitative Polymerase Chain Reaction (qPCR), Reverse Transcriptase qPCR (RT-qPCR), and microarrays	qPCR: abundance of a specific gene RT-qPCR: expression level of a specific gene Microarray: abundance of multiple genes	The potential for biodegradation of a contaminant in the groundwater plume	- Metals and humic substances may affect results. Specific protocols for soil samples must be used. - Microbes that grow attached to the aquifer matrix may be underrepresented. Passive microbial sampling devices may be used, but perfect representation is not guaranteed.
